# Cytotoxic Activity of Silyl- and Germyl-Substituted 4,4-Dioxo-3a,6a-Dihydrothieno[2,3−d]isoxazolines-2

**DOI:** 10.1155/MBD.2000.63

**Published:** 2000

**Authors:** E. Lukevics, P. Arsenyan, I. Shestakova, O. Zharkova, I. Kanepe, R. Mezapuke, O. Pudova

**Affiliations:** Latvian Institute of Organic Synthesis Aizkraukles 21 Riga LV-1006 Latvia

## Abstract

The [2+3] dipolar cycloaddition of nitrile oxides to the double C = C bonds of thiophene-1, 1-dioxides leads to formation of the fused isoxazolines-2 (**1**, **2**). Tumor growth inhibition of these compounds strongly depends on the nature of group IV A element increasing from slightly active *tert-butyl* derivatives to silicon and germanium containing analogues. The products of benzonitrile oxide cycloaddition have greater cytotoxic effect than the compounds obtained from the cycloaddition reaction of 2, 5-disubstituted thiophene-1, 1-dioxides with acetonitrile oxide. Fused silyl substituted isoxazolines-2 are stronger NO-inducers than their germyl and *tert-butyl* analogues.


					CYTOTOXIC ACTIVITY OF SILYL- AND GERMYL-SUBSTITUTED
4,4-DIOXO-3a,6a-DIHYDROTHIENO[2,3-d]ISOXAZOLINES-2
E. Lukevics*, P. Arsenyan, I. Shestakova, O. Zharkova, I. Kanepe,
R. Mezapuke, and O. Pudova
Latvian Institute of Organic Synthesis, Aizkraukles 21, Riga, LV-1006, Latvia
ABSTRACT
The [2+3] dipolar cycloaddition of nitrile oxides to the double (3=(3 bonds of
thiophene-l,l-dioides leads to formation of the fused isoxazolines-2 (1, 2). Tumor growth
inhibition of these compounds strongly depends on the nature of group IV A element
increasing from slightly active ert-butyl derivatives to silicon and germanium containing
analogues. The products of benzonitdle oxide cycloaddition have greater cytotoic effect
than the compounds obtained from he cycloaddition reaction of 2,5-disubstituted
thiophene-l,l-dioxides with acetonitrile oxide. Fused silyl substituted isoxazolines-2 are
stronger NO-inducers than their germyl and tert-butyl analogues.
INTRODUCTION
The interest in silyl substituted thiophene-l,l-dioxides stems from the fact that they
are useful synthetic intermediates for the preparation of various types of organic compounds
by Diels-Alder cycloaddition [1], amine induced ring-opening reaction [2], or coupling of
bromothiophene-l,l-dioxides with thienyl stannanes in the presence of a palladium (0)
catalyst [3]. It has been shown that unsubstituted thiophene-l,l-dioxide prepared in situ is a
quite reactive dipolarophile in the [2+3] cycloaddition reactions with N,o-diphenylnitrone
[4], benzonitrile [4, 5] and mesitonitrile [4, 5] oxides yielding mono- and diisoxazolines-2 and
N-substituted isoxazolidines. Moreover, our recent studies indicate that silyl- and germyl-
containing isoxazolines have gained a great deal of attention as compounds possessing a
wide spectrum of the biological properties. The vasodilating, anticoagulant and
cardioprotective activity of 5-Si-(Ge)substituted isoxazolines-2 has been studied in vitro and
in vivo [6, 7]. The most active isoxazoline -3-(5"-triethylgermyl-3"-isoxazolino)pyridine
hydrochloride protected the heart from rhythm disturbances and lethality during ischemia-
reperfusion [7]. It has been shown that silylisoxazolines-2 are more potent in protection
against hypoxia and corazole convulsions than germanium analogues. However,
germylisoxazolines-2 are stronger tumor growth inhibitors and NO-inducers than their silicon
analogue [8].
This work presents the results of cytotoxic activity for fused isoxazolines-2 bearing a
group 14 element as substituent (1, 2) in function of the nature of the group 14 element.
-0\
N
O\
N
Me3M.CH3 Me3MS
02
R
02
1 2
MATERIALS AND METHODS
CHEMISTRY
Seven tert-butyl-, trimethylsilyl-, and trimethylgermyl-substituted 4,4-dioxo-3a,6a-
dihydrothieno[2,3-d]isoxazoline-2 1 and 2 (Table 1)were prepared by the [2+3] dipolar
63
Vol. 7, No. 2, 2000 Cytotoxic Activity ofSilyl-and Germyl-Substituted 4,4-dioxo-3a
,6a-Dihydrothieno[2,3-d]Isoxazolines-2
cycloaddition of aceto- and benzonitrile oxides to 2,5-disubstituted thiophene-l,l-dioxides.
Their synthesis and characterization are given in ref. [9].
_,,/0\N
Me3M M'Me3 Me3M %, \R
02 02
M, M'=C, Si, Ge; R=Me, Ph; R'=H, Me3Ge
Table 1. Investigated Me3C, MeSi,
dihydrothieno[2,3-d]isoxazolines-2
Me3Ge substituted 4,4-dioxo-3a,6a-
Compound
4,4-dioxo-3 methyl"5-tert-butyl-3a,6a-
dihydrothieno[2,3-d]isoxazoline-2
4,4-dioxo-3-methyl-3a-trimethylgermyl-5-tert-butyl-
3a,6a-d ihydrothieno[2,3-d]isoxazoline-2
4,4-dioxo-3-methyl-3a-trimethyigermyl-5-
trimethylsilyl-3a,6a-d hydrothieno[2,3-
d]isoxazoline-2
4,4-dioxo-3-methyI-3a, 5-b s trimethyIgermyI)-
3a,6a-d hydrothieno[2,3-d]isoxazo ne-2
4,4-dioxo-3-phenyI-5-tert-butyI-3a,6a-
dihydrothieno[2,3-d]isoxazoline-2
4,4-dioxo-3-phenyI-5-trimethy silyI-3a,6a-
dihydrothieno[2,3-d]isoxazoline-2
4,4-dioxo-3-phenyI-5-trimethyIgermyI-3a,6a-
di,hydr0thieno[2,3,d]isoxazoline-2
'Type M R
la (3' H
Yield
(%)
80
lb C Me3Ge 58
lc Si Me3Ge 45
ld Ge Me3Ge 67
2a C 84
2b Si 77
2c Ge 85
IN VITRO CYTOTOXITY ASSAY
Monolayer cells lines were cultivated for 72 h in DMEM standard medium without an
indicator and antibiotics. After the ampoule was defreezed not more than four passa4ges were
performed. The control cells and cells with tested substances in the range of 2-5 10 cell/mL
concentration (depending on line nature)were placed on a separate 96 wells plates.
Solutions containing test compounds were diluted and added in wells to give the final
concentrations of 50, 25, 12.5, and 6.25 #g/mL Control cells were treated in the same
manner only in the absence of test compounds. Plates were cultivated for 72 h. A quantity of
survived cells was determined using crystal violet (CV) or 3-(4,5-dimethylthiazol-2-yl)-2,5-
diphenyltetrazolinium bromide (MTT) coloration which was assayed by multiscan
spectrofotometer. The quantity of alive cells on control plate was taken in calculations for
100% [10, 11]. Concentration of NO was determined according to [10].
RESULTS AND DISCUSSION
Potential cytotoxic activity of synthesized fused isoxazolines 1 and 2 was tested in
vitro on four monolayer tumor cell lines: MG-22A (mouse hepatoma), HT-1080 (human
fibroblastoma), B16 (mouse melanoma), Neuro 2A (mouse neiroblastoma). Concentrations
providing 50% of tumor death effect were determined according to the known procedure [12]
using 96 well plates.
The experimental evaluation of cytotoxicity properties is presented in Table 2. A
preliminary analysis of the structure-activity relationship for the cytotoxic action clearly
indicates the strong influence of the MeM (M=C, Si, Ge) group in position 5 of fused
isoxazolines 1 and 2. Derivatives bearing tert-butyl substituent (la,b and 2a) have a slight
cytotoxic effect (> 10 #g/mL). The substitution of the tert-butyl group by trimethylsilyl or
64
E. Lukevics et al. Metal-Based Drugs
A
oO {D 0 0
65
Vol. 7, No. 2, 2000 Cytotoxic Activity ofSilyl-and Germyl-Substituted 4,4-dioxo-3a
,6a-Dihydrothieno[2,3-d]Isoxazolines-2
trimethylgermyl ones leads to considerable increase of cytotoxicity. It must be noted that the
activity of silicon- and germanium-containing compounds (1 and l d) depends on the tumor
type. 5-Trimethylsilyl-substituted fused isoxazoline le is more active than the germanium
analogue l d in tests on HP-1080 and MG-22A cell lines. However, the germanium
compound ld has greater cytotoxic effect on Neuro 2A and B16 cell lines than the silicon
derivative lc. Comparison of the tumor growth inhibition for derivatives 1 and 2 shows a
higher activity of the condensed isoxazolines 2 containing a phenyl group in position 3 with
respect to 4,4-dioxo-3-methyl-3a-trimethylgermyl-5-MeM-3a,6a-dihydrothieno[2,3-
d]isoxazolines l b-d. Silyl- and germyl-substituted fused isoxazolines have a medium NO-
induction ability, 4,4-dioxo-3-phenyl-5-trimethylsilyl-3a,6a-dihydrothieno[2,3-d]isoxazoline-2
(2b) being the most active (250% in the MG-22A test).
ACKNOWLEGMENT
We are grateful to Latvian Taiho Foundation for financial support.
REFERENCES
1. A. R. M. Donovan, M. K. Shepherd, Tetrahedron Lett., 35 (1994)4425.
2. S. Gronowitz, A.-B. H0rnfeldt, E. Lukevics, O. Pudova, Synthesis, (1994)40.
3. G. Barbarella, L. Favaretto, G. Sotgiu, M. Zambianchi, L. Antolini, O. Pudova, A.
Bongini, J. Org. Chem., 63 (1998) 5497.
4. A. Bened, R. Durand, D. Pioch, P. Geneste, J. P. Declerq, G. Germain, J. Rambaud, R.
Roques, J. Org. Chem., 46 (1981) 3502.
5. F. M. Albini, P. Ceva, A. Mascherpa, E. Albini, P. Caramella, Tetrahedron, 38 (1982)
3629.
6. E. Lukevics, M. Veveris, V. Dirnens, Appl. Organomet. Chem., 11 (1997) 805.
7. E. Lukevics, P. Arsenyan, M. Veveris, Metal Based Drugs, 5 (1998) 251.
8. E. Lukevics, P. Arsenyan, S. Germane, I. Shestakova, Applied Organomet. Chem., 13
(1999) 795.
9. E. Lukevics, P. Arsenyan, S. Belyakov, J. Popelis, O. Pudova, Organometallics, 18 (1999)
3187.
10. D.J. Fast, R.C. Lynch, R.W. Leu, J. Leuckocyt. Biol., 52 (1992) 255.
11. P.J. Freshney, Culture of Animal Cells (A Manual of Basic Technique), Wiley-Liss, New
York, 1994, pp. 296-297.
12. R. J. Riddell, R. H. Clothier, M. Fd. Balls, Chem. Toxicol., 24 (1986) 469.
Received: January 21, 2000 Accepted: February 1, 2000
Received in revised camera-ready format" February 2, 2000
66

				

